# Aspirin Responsiveness in a Cohort of Pediatric Patients with Right Ventricle to Pulmonary Artery Conduits and Transcatheter Valve Replacement Systems

**DOI:** 10.1007/s00246-024-03449-1

**Published:** 2024-03-15

**Authors:** Sean T. Kelleher, Irene E. Regan, Dermot Cox, Kathryn Shaw, Orla Franklin, Damien P. Kenny, Kevin P. Walsh, Colin J. McMahon

**Affiliations:** 1 Department of Paediatric Cardiology, Children’s Health Ireland at Crumlin, Dublin, Ireland; 2 Department of Coagulation/Haematology, Children’s Health Ireland at Crumlin, Dublin, Ireland; 3 School of Pharmacy and Biomolecular Sciences, Royal College of Surgeons Ireland, Dublin, Ireland; 4 Department of Paediatric Pharmacy, Children’s Health Ireland at Crumlin, Dublin, Ireland; 5 School of Medicine, University College Dublin, Belfield, Dublin 4, Ireland; 6 School of Health Professions Education (SHE), Maastricht University, Maastricht, The Netherlands; 7 National Children’s Research Centre, Children’s Health Ireland, Dublin, Ireland

**Keywords:** Aspirin, Conduit, Transcatheter valve replacement, Platelet, Resistance, Thrombosis

## Abstract

The aim of this study was to determine the rate of aspirin responsiveness in a cohort of pediatric patients with in situ xenograft valved right ventricle to pulmonary artery (RV-PA) conduits and/or transcatheter valve replacements (TVR). Aspirin is routinely prescribed to these patients. Optimizing anti-platelet therapy could promote valve longevity and reduce the risk of infective endocarditis in this at-risk group. This was a prospective, observational study. Patients were recruited from both ward and outpatient settings. Patients were eligible if under 18 years and taking aspirin. Non-response to aspirin was defined as > 20% platelet aggregation using light transmission platelet aggregometry (LTA) and < 50% platelet inhibition by thromboelastography with platelet mapping (TEGPM). Participants were invited to provide a confirmatory sample in cases of aspirin resistance and dose adjustments were made. Thirty patients participated. Median age was 9 years (2 months to 18 years). The majority (93%) had complex right ventricular outflow tract pathology. 13 (43%) had an RV-PA conduit and 24 (80%) had a TVR, with valve situated in conduit in 7 (23%) cases. Rate of aspirin non-response on initial testing was 23% (*n* = 7/30) with median LTA 74.55% (60–76%) and TEG 13.25% (0–44%) in non-responders. Non-responders were more likely to be under 1 year. Two patients required dose increases and one patient non-adherence to dose was identified. Four patients on repeat testing were responsive to aspirin by laboratory tests. The rate of aspirin non-response on laboratory testing in this cohort of patients was 23% and resulted in therapeutic intervention in 10%.

## Introduction

Aspirin possesses a myriad of biological effects, one of its most pertinent being its ability to inhibit platelet aggregation. Achieved through its irreversible inhibition of the cyclo-oxygenase-1 (COX-1) enzyme, aspirin limits the access of arachidonic acid to a key site on COX-1, attenuating the downstream production of thromboxane A2 (TXA2), a powerful platelet aggregator. The effect of aspirin on the arachidonic acid pathway lasts the lifespan of the platelet [1]. There are, however, numerous other pathways of platelet activation including the ADP pathway, upon which the P2Y_12_ inhibitor clopidogrel acts [2]. Aspirin non-responsiveness is defined as the inability of aspirin to decrease the production of thromboxane A2 and prevent platelet aggregation in a laboratory test. It may arise through a variety of mechanisms including under-dosing, drug–drug interactions, increased platelet turnover, and genetic polymorphisms [3].

The first report of bovine jugular valved right ventricle to pulmonary artery (RV-PA) conduits used in animal studies dates from 1992 [4]. Since then, valved RV-PA conduits, including the Contegra® (bovine) and Hancock® (porcine) conduits, have become widely used surgical options to treat complex forms of right ventricular outflow tract obstruction in the paediatric population [5, 6]. Over time, such conduits may become stenotic or develop valvar insufficiency requiring replacement. Transcatheter pulmonary valve replacement (TPVR) with stent-mounted xenograft balloon expandable valve systems has in recent years offered an alternative to surgical conduit replacement. The first such valve, the Melody®, was approved by the FDA in 2010 and is a bovine jugular venous (BJV) valve system [7]. Additionally, there is now widespread experience with both the Edwards SAPIEN® valve and the Venus-P system, stent-mounted xenograft bioprosthetic valves with leaflets composed of bovine and porcine pericardium, respectively [8, 9]. Increasingly, transcatheter valve replacement (TVR) is being utilized in the mitral and tricuspid positions [10, 11]. Mid- and long-term outcomes post-TPVR are favorable with survival 91% and freedom from intervention 75% at 8 years [12]. However, there is increasing concern regarding the cumulative risk of infective endocarditis (IE) in patients with xenograft bioprosthetic heart valves with an annualized incidence of IE post-TPVR of 2.2 per 100 patient years [13]. Evidence suggests that the incidence of infective endocarditis (IE) with the use of BJV systems is higher than with other valve types (homograft and bioprosthetic valves) [14].

While aspirin appears to be routinely prescribed among these patients, there are a paucity of prescribed guidelines for the optimal use of anti-coagulant and anti-platelet agents in patients with an RV-PA conduit, TPVR, or TVR in the mitral or tricuspid positions. A recent survey of interventionalists demonstrated that post-TPVR, 100% prescribed aspirin, but there was a wide variation in dosing and use of additional agents [15]. While thromboembolic events are certainly low in this group [16], a recent series of explanted valved conduits demonstrated very high rates of sub-clinical thrombus adherent to the valve sinuses, the presence of which may contribute to valve failure and act as a nidus for IE [17]. Furthermore, abrupt discontinuation of aspirin has been highlighted as a potential risk factor for the development of IE in patients with Melody® valve in situ [18].

There have been calls to further clarify the role of aspirin and anti-platelet agents in mitigating the risk of IE and valve failure [13, 17]. There have been no previous studies of failure to respond to aspirin in this context and most practitioners do not routinely test for it [15]. Our aim was to determine the rate of aspirin non-response in a cohort of pediatric patients with in situ xenograft valved conduits and TVR systems.

## Methods

### Patient Selection

This study was approved by The Research and Ethics Committee of Children’s Health Ireland at Crumlin, Dublin, Ireland (GEN/580/17). This study formed part of a larger study on aspirin response in pediatric patients with congenital heart disease. Written informed parental consent was obtained for each participant. All patients were attending cardiac services at Children’s Health Ireland at Crumlin. Recruitment took place between January 2019 and June 2023. Patients were included if they had an in situ RV-PA xenograft valved conduit or a TVR system and were taking aspirin. Exclusion criteria are listed in Table 1.

**Table 1:** Exclusion criteria

Age > 18 years
Known coagulation disorder
Abnormal platelet function
Thrombocytopenia < 150 × 10^9^/L
Hemoglobin < 100 g/L
Active endocarditis

Patients were prospectively enrolled from both ward and outpatient settings.

Aspirin was dosed at 3–5 mg/kg, up to a maximum of 75 mg. Institutional practice is to prescribe to the nearest quarter of a 75-mg tablet due to the inaccuracy of dispersion techniques with such preparations [19].

### Testing

Aspirin response was tested a minimum of 2-h post first dose of aspirin, with most patients on established aspirin therapy at time of testing. Arachidonic-induced platelet aggregation was abolished in the majority 2–6-h post-dose in previous adult studies [20]. If non-response was detected, adherence and dose timing were discussed. Participants were invited to provide a confirmatory sample. Dose adjustments, if deemed appropriate, were made in consultation with the primary physician and typically increased in quarter tablet (of 75 mg) increments. Aspirin response was measured using two separate tests described below: Thromboelastography with Platelet Mapping (TEGPM) and Light Transmission Platelet Aggregation (LTA). For patients under the age of 2 years, TEGPM alone was performed due to the phlebotomy requirements for completion of both tests.

### Thromboelastography with Platelet Mapping (TEGPM)

TEGPM was performed using a platelet mapping assay on the TEG® 5000 analyser platform. This gives a quantitative analysis of platelet function based on the formation, strength, and degradation of clots in whole blood. It allows for the contribution of aspirin to be assessed through the addition of arachidonic acid (AA) to determine the response of the TXA2 receptor when compared with standard samples from the index patient. Three assays were performed. The first was performed by adding whole blood to kaolin and measuring TEG on this kaolin-activated blood to derive maximal clot strength (MAThrombin) in the standard TEG fashion. The contribution of fibrin to clot strength (MAFibrin) was assessed through the addition of reptilase and factor XII and measured on a second TEG cup. Finally, arachidonic acid (AA) and Activator F were added to a sample in a third TEG cup to assess the contribution of the COX-1 pathway (MA AA). The percentage platelet inhibition was calculated using the equation 100 − {(MA AA − MA Fibrin)/(MA Thrombin − MA Fibrin) × 100} [21]. Non-responsiveness to aspirin was defined as platelet inhibition < 50%. To ensure consistency across assays and as a quality control measure, the MA AA was repeated using the same agonist AA as employed in the LTA-AA assay. This agonist was substituted for the kit AA and the MA AA analyzed again as initially.

TEG-PM has a sensitivity of 100% and specificity of 92% when using a cut-off of 50% platelet inhibition [21–23]. TEG-PM also offers some advantages over other point of care as many are strongly dependent on platelet count and provides information on clotting factors, including fibrinogen [24].

### Light Transmission Platelet Aggregometry (LTA)

LTA was performed on platelet-rich plasma samples by placing the sample between the light source and the photocell. Arachidonic acid was added to the sample in order to activate platelets. Aspirin non-response was defined as platelet aggregation in response to arachidonic acid of > 20%.

LTA using the agonist AA (LTA-AA) is considered one of the gold standard tests for the detection of patients resistant to aspirin [25]. In our institution, we have shown good correlation between this assay and another gold standard test, serum thromboxane B2 (TXB2), which is possibly more specific to evaluate the inhibitory effect of aspirin on platelets [26, 27]. In our institution, we find the serum TXB2 to be a more time-consuming test and usually analyzed in batches, while analysis by LTA-AA has the advantage of faster turn-around times with good sensitivity and specificity 100% and 95.6%, respectively, when using a cut-off of 20% aggregation [28].

### Statistical Analysis

Baseline and demographic data were summarized for the enrolled patients. Normally distributed continuous data are expressed as mean ± SD, non-normally distributed variables were expressed as median (minimum–maximum). Categorical variables were expressed as percentages. Comparison of characteristics between responders and non-responders was performed using the Mann–Whitney *U* test for continuous variables, and Fisher exact test for categorical data. Statistical analysis was performed using GraphPad Prism version 10.0.0.

## Results

### Demographic Characteristics

In total, 30 patients were recruited to participate. Demographic data are listed in Table 2. Median age was 9 with a broad range of age groups represented with the youngest participant aged 2 months. The most common single diagnosis was pulmonary atresia (PA), a heterogeneous group which included patients with intact ventricular septum, ventricular septal defect, and major aorto-pulmonary collateral arteries (MAPCAS) who had undergone biventricular repair. Also included were patients with TVR in the mitral (*n* = 1, Melody®) and tricuspid (*n* = 1, Edwards SAPIEN®) positions.

**Table 2:** Demographics and patient characteristics

Demographic/characteristic	Total *n* = 30
Age (years)	9 (0.17–18)
<1 year	2 (7)
1-4 years	5 (17)
5-10 years	9 (30)
11-18 years	14 (46)
Sex	
Female	19 (63)
Male	11 (37)
Primary Diagnosis	
Pulmonary atresia	12 (40)
o w/ VSD, MAPCAS	o 6 (20)
o w/ VSD	o 4 (13)
o w/ ccTGA	o 2 (7)
Tetralogy of Fallot	11 (37)
Complex TGA	3 (10)
Truncus Arteriosus	2 (7)
Pulmonary stenosis	1 (3)
Mitral regurgitation	1 (3)
RV-PA Conduit	13 (43)
Contegra	11 (37)
Hancock	2 (7)
Transcatheter Valve Replacement Systems	24 (80)
Melody	- 19 (63)
Edwards SAPIEN	- 4 (13)
Edwards Perimount	- 1 (3)
Valve Position	
Native/patched RVOT	- 15 (50)
RV-PA Conduit	- 7 (23)
Mitral Position	- 1 (3)
Tricuspid Position	- 1 (3)
Testing setting	
Outpatient	- 20 (67)
Post-operative period	- 4 (13)
Post-interventional procedure	- 6 (20)
Lifetime history of infective endocarditis	4 (13)
Median aspirin dose/kg (minimum to maximum)	2.93 mg/kg/day (0.54-5.35)

Values are *n*(%), unless otherwise stated. TGA = Transposition of the great arteries, RV-PA = right ventricle to pulmonary artery, RVOT = right ventricular outflow tract

### Test Results

There were 30 initial tests and 9 instances of repeat testing. In the majority of cases (82%), both TEGPM and LTA were performed. Two patients had two repeat tests performed. There was good agreement between the tests. There was one occurrence of discordant results in which the patient was classified as being responsive by LTA (Aggregation: 10.4%) and having a borderline low response by TEGPM (Inhibition: 30.1%).

Seven (23%) of patients demonstrated non-response to prescribed aspirin therapy as defined by TEGPM and/or LTA on initial testing. Laboratory test results are detailed in Table 3. There was no statistically significant difference between the median age of the two groups, but non-responders were more likely to be under 1 year. There were no statistically significant differences between the baseline characteristics and testing context between the two groups (out-patient vs. post-operative or post-procedural).

**Table 3:** Laboratory test results

Test parameter	Result
Testing points	Total *n* = 39
TEG-PM	37 (95)
LTA	34 (87)
Both	32 (82)
Non-responsive on initial testing	7 (23)
Discordant results	1 (3)
LTA responsive (median percentage aggregation)	8.45% (0.7–20%)
TEGPM responsive (median percentage inhibition)	100% (64–100%)
LTA non-responsive (median percentage aggregation)	74.55% (60–76%)
TEGPM non-responsive (median percentage inhibition)	13.25% (0–44%)
Test timing post-dose (mean)	10.3 h

Values are *n*(%), unless otherwise stated, ranges are (minimum to maximum), TEG-PM = Thromboelastography with platelet mapping, LTA = light transmission platelet aggregometry

### Characteristics of Patients Demonstrating Aspirin Non-response

The baseline characteristics and postulated etiology of the 7 patients demonstrating inadequate response to aspirin (non-response) on initial testing are outlined in Table 4. Where there was an initial abnormal test result, patients were invited to return for repeat confirmatory testing. In case 5, repeat testing was not performed, and aspirin dose was increased at the discretion of the attending physician.

**Table 4:** Outcomes of patients demonstrating aspirin non-response

No.	Diagnosis	Conduit/Valve	Age (yrs)	Weight (kg)	Test setting	Dose (mg)	TEGPM (%) Cut-off for adequate response >50%	LTA (%) Cut-off for adequate response <20%	Test repeated	Repeat result	Outcome
1	Pulmonary atresia	RV-PA Conduit	2	18	OPD	75	26.5	76	yes	R	Adherence discussed
2	Pulmonary atresia	RV-PA Conduit	0.75	8.5	IP	37.5	0	–	yes	R	Attributed to post-operative period
3	Pulmonary atresia	TVR in RV-PA Conduit	12	50	OPD	75	43	60	yes	R	No cause identified
4	Pulmonary stenosis	TVR (pulmonary)	2	14	OPD	75	0	74	yes	R	Dose timing
5	Tetralogy of Fallot	TVR (pulmonary)	11	–	OPD	75	0	75.1	no	-	Dose increase
6	Truncus Arteriosus	RV-PA Conduit	0.17	4	OPD	18.75	44.5	–	yes	NR	Dose increase.
7	Complex TGA	TVR (tricuspid)	12	42	OPD	75	30.4	10.4	yes	R	Resolution of discordant results on repeat

TEGPM = thromboelastography with platelet mapping, LTA = light transmission platelet aggregometry, RV-PA = Right ventricle to pulmonary artery, TVR = Transcatheter Valve Replacement , TGA = transposition of the great arteries, R = responsive, NR = Non-responsive, OPD = outpatient, IP = Inpatient

Six patients had repeat testing performed, and in 5 of the 6 cases, the repeat test demonstrated adequate response to aspirin. In case 1, non-adherence with the prescribed dose was identified. In case 2, the initial sample was taken in hospital in the post-operative setting. Repeat testing beyond the post-operative period demonstrated response to aspirin. In case 3 no cause was identified. In case 4, test timing may be implicated with the previous dose had been taken 24 hours before the sample. In case 6, aspirin non-response was identified on an appropriate dose on sequential testing that resolved on dose increase. In case 7, there were discordant results which was no longer apparent on repeat (described below).

## Discussion

### Summary

In this prospectively recruited observational series, this is the first time that the rate of aspirin non-responsiveness among patients with TVR systems and xenograft valved conduits has been reported. An initial rate of aspirin non-responsiveness of 23% was identified in this group. However, in 17% (*n* = 5) of patients repeat confirmatory testing at a separate time point demonstrated adequate response to aspirin, with non-adherence to the correct dose of aspirin confirmed in 3% (*n* = 1). The true rate of aspirin non-responsiveness requiring dose increase in this group may be as low as 6.7%. In 10% (*n* = 3), testing for aspirin response resulted in a therapeutic intervention consisting of dose increases in two cases and medication education in another. Non-responders were more likely to be under the age of 1 (29% vs 0%). Of the two non-responders under 1, one was in the post-operative period. Increased vigilance in this age group may be advised. Agreement between tests was strong, which if consideration was made to adoption of testing to routine practice would decrease phlebotomy requirements. Repeat testing is recommended for instances of discordance which can occur due to differences in methodologies, sensitivity, and specificity of the assays and cut-off values.

### The Post-operative Period

Previous studies of pediatric patients with congenital heart disease have demonstrated rates of aspirin non-responsiveness between 10 and 50%. Some of the variation in reported rates may be attributable to the context in which testing was performed with previous literature largely focusing on the post-operative period, which appears to be a high-risk period for decreased response [29–32]. The reason for decreased aspirin response post-operatively is likely multi-factorial. In adult series, cardiopulmonary bypass appears to impact rates of aspirin non-responsiveness, with lower rates in non-bypass surgery [33]. Mechanistically, recent published work by this group, demonstrates that in paediatric patients post cardiac surgery, high platelet turnover results in high levels of immature platelets with increased thrombotic potential and inadequate response to aspirin [34]. Inadequate response to aspirin in post-operative paediatric patients with CHD, has been demonstrated to be associated with higher mortality from thrombotic events [30]. Furthermore, testing for aspirin response in the post-operative period and intervening with dose increases has been shown to lower rates of thrombotic events [29]. Whether the period post percutaneous procedures confers any additional risk is under-explored. However, none such patients experienced aspirin non-responsiveness in this study.

### Patient Age and Non-response to Aspirin

Neonatal patients possess an immature coagulation system with low levels of anti-thrombotic factors [35]. Significantly higher rates of thrombosis are observed in neonates and young infants when compared to older children [36]. Rates of aspirin non-response of up to 80% have been identified, in the early post-operative period in neonates and young infants following first stage surgical palliation for single ventricle physiology [37]. It is notable that both infants under the age of 1 in this study were non-responsive on initial testing.

### Adherence, Pharmacokinetic Resistance and Pharmacodynamic Resistance

The majority (67%) of patients in this study were well out-patients. Adherence with medication has been implicated in adult studies on aspirin resistance [38], but adherence may be underestimated in the paediatric population and is an important consideration in the out-patient setting [39]. In adult series, pharmacokinetic resistance is frequently identified in cases of aspirin non-response. Pharmacokinetic resistance signifies low plasma levels of aspirin despite adequate intake. Frequently prescribed enteric-coated preparations can have significant effects on bioavailability and are the norm unless specified by the prescribing physician [40–43]. The authors, therefore, recommend dispersible aspirin for use in paediatric patients. Concurrent use of NSAIDs, and proton-pump inhibitors can also affect bioavailability and their use is widespread in paediatrics [15]. Pharmacodynamic resistance in which there is adequate bioavailability of aspirin but no inhibition in-vitro appears to be rare, especially that which cannot be overcome by dose increase [3, 29, 40, 41, 43]. While, data are lacking in paediatrics, one adult study in an out-patient setting reduced the rate of aspirin non-response from 25 to 2% through adherence (direct observation) and use of non-enteric coated aspirin. The remaining 2% of patients responded to doubling of aspirin dose. A strength of this study is that confirmatory testing was requested prior to therapeutic changes and interestingly a proportion of patients initially deemed non-responsive were responsive on repeat.

### Approach to Aspirin Non-response and Implications

**Fig. 1:**
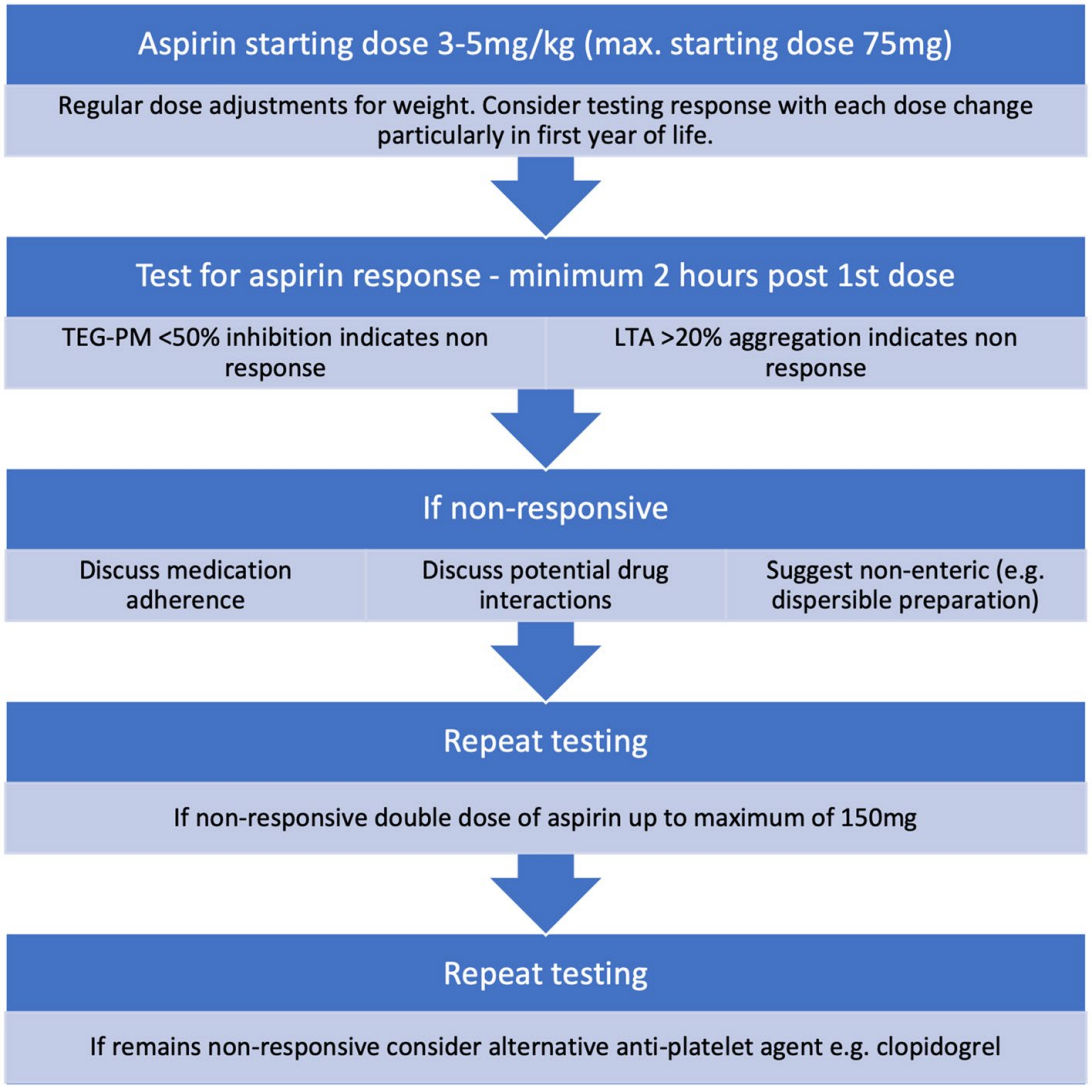
Proposed aspirin responsiveness testing algorithm

The authors advocate increased vigilance in the post-operative period particularly in neonates and young infants when considering aspirin non-response, with careful consideration paid to dose increases. Within the outpatient setting, consideration should be paid to aspirin preparation, adherence and drug interactions. Dose adjustment may be required to achieve response and rarely switching to an alternative anti-platelet agent. A proposed algorithm is detailed in Fig. 1. In the context of TVR systems and xenograft valved conduits, the role that aspirin non-responsiveness may play in valve longevity and risk of IE has not been demonstrated. This is the first study to explore the rate of aspirin non- responsiveness in this context. Further studies are necessary to explore the link between inadequate response and adverse outcomes, and indeed whether aspirin alone is sufficient to mitigate this risk.

### Limitations

This is a small, single-centre observational study, without control group for comparison. This limits the inferences that can be drawn. The patient group is heterogenous in terms of age, diagnosis, valve position and test setting. While our study demonstrated statistically significant higher rates of non-response in the under 1 age category (similar to previous work on the topic), it is notable that only two children under the age of 1 participated. This study was not powered to assess whether aspirin non-responsiveness was associated with adverse outcomes in this group of patients. Not assessed by this study is the link between genetic polymorphisms and inadequate response to aspirin. Gene polymorphisms in dual antiplatelet therapy may be associated with the presence of leaflet thickening after transcatheter aortic valve replacement [44], but this has not been demonstrated post TPVR.

Notable in this series is the rate of prior IE of 13% indicating that this is certainly a high-risk group. However, this is not indicative of IE within the study period and inferences between aspirin response and IE could not be drawn.

The variable response to aspirin in some patients over time is not fully understood. False positive test results need to be considered. However, in an effort to mitigate against this, all patients were analysed by employing two assays where possible. TEG data was confirmed using surrogate arachidonic acid, the same arachidonic acid used in the LTA-AA assay. All analysis was repeated on the same samples using the initial assays to confirm results. The interpretation of results of patients who demonstrate initial non-response with normal results on follow up testing is therefore more difficult. Postulated aetiologies have been listed in Table 4. The authors suggest that fluctuations in response may be due to non-adherence, pharmacokinetics (typically attributed to enteric coated preparations or drug-drug interactions) or pharmacodynamic effects. These issues can typically be overcome with the measures outlined within Fig. 1.

While there was no significant difference in test timing between responders and non-responders (Table 5), the effect of test timing in a paediatric population is not well characterised. In a small series of adult patients, on sequential testing over a 24-h period with LTA, Henry et al. demonstrated that the proportion of patients with in vitro resistance was lowest at 2 h [45]. One patient on established aspirin therapy was tested before their regular once-daily dose i.e. 24-h post dose, with normal results on re-testing. Optimal test timing may be 2–12 h post dose [28], which is contrary to the hypothesis that aspirin effect potentiates for the life cycle of the platelet. Where possible, the authors recommend test-timing fall within this interval, while acknowledging this may be challenging particularly in paediatric patients. Consideration of dosing interval was outside the scope of this study.

**Table 5:** Comparison of Characteristics of Responders and Non-responders

	Non-responders (*n* = 7)	Responders (* n* = 23)	*p*-value
Age* (years)	2.75 (0.2–13)	10 (2–18)	0.076
Under 1 year	2 (29)	0 (0)	0.048
Weight* (kg)	16 (4–50)	27 (6–69)	0.133
Normalized dose* (mg/kg)	4.29 (1.5–5.35)	2.63 (0.54–4.87)	0.066
Post-op	1 (14)	3 (13)	>0.99
Post-procedure	0 (0)	6 (26)	0.29
Test timing post-dose (mean)*	10.66 h	10.18 h	0.95

Values are Median (minimum to maximum), *n*(%), unless otherwise stated. *Parameters assessed using Mann–Whitney U Test and remaining parameters assessed used Fisher’s Exact test.

## Conclusions

The rate of aspirin non-responsiveness amongst paediatric patients with transcatheter valve replacement systems or xenograft valved conduits was 24% and testing for aspirin response should be considered in these patients. However, only 6.7% of patients required dose increases. The authors recommend reviewing aspirin dose regularly and up-titrating dose for weight, with consideration for responsiveness testing with dose increases particularly under the age of 1 year. Increased vigilance for non-response in the post-operative period has been previously shown to reduce thrombotic events [29]. Where non-response is identified, confirmation of adherence and additional medications should be reviewed. The authors advocate for the prescription of non-enteric coated preparations. A testing algorithm has been proposed (Fig. 1).

Whether aspirin possesses a protective effect against the development of IE and valve failure in these patients remains to be fully elucidated. Echoing the call from previous literature, there is a need for clear guidance on the optimal anti-platelet regimen in this patient cohort [13, 15, 17].

## Data Availability

Data available on request.
